# Supplementary material for: Untangling Synergistic Effects of Intersecting Social Identities with Partial Information Decomposition
Source: Entropy (Basel). 2022 Sep 28;24(10):1387. doi: 10.3390/e24101387 (PMC9611752; doi:10.3390/e24101387)
Supplement: Supplementary file 1 [file entropy-24-01387-s001.zip › Supplementary (SI PDF + Script.zip)/Scripts.zip!/scripts/pid_bars.pdf]

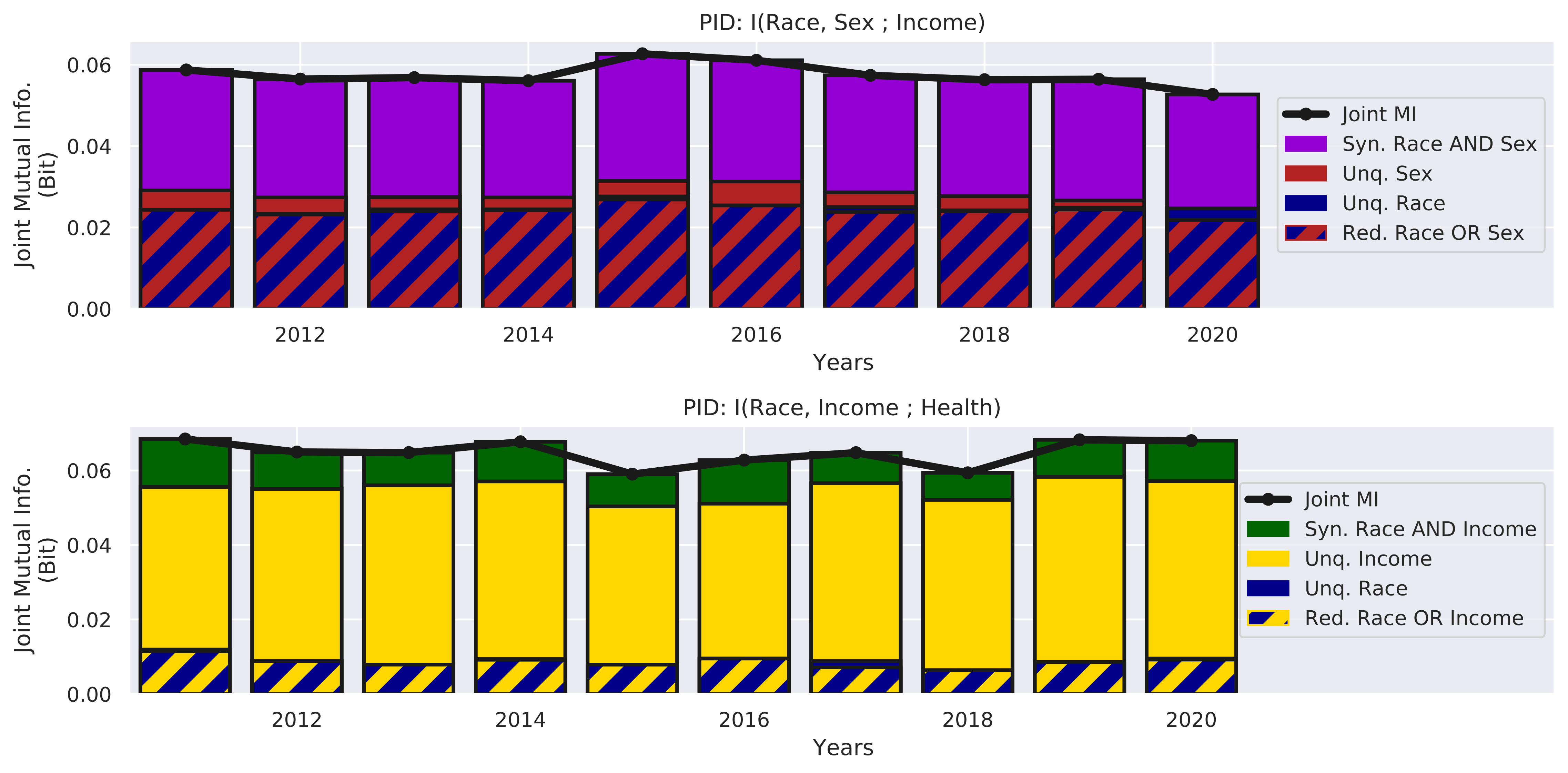
PID: I(Race, Sex ; Income)
Joint Mutual Info. (Bit)
0.00
0.02
0.04
0.06
2012
2014
2016
2018
2020
Years
Joint MI
Syn. Race AND Sex
Unq. Sex
Unq. Race
Red. Race OR Sex
PID: I(Race, Income ; Health)
Joint Mutual Info. (Bit)
0.00
0.02
0.04
0.06
2012
2014
2016
2018
2020
Years
Joint MI
Syn. Race AND Income
Unq. Income
Unq. Race
Red. Race OR Income
