# Supplementary material for: Untangling Synergistic Effects of Intersecting Social Identities with Partial Information Decomposition
Source: Entropy (Basel). 2022 Sep 28;24(10):1387. doi: 10.3390/e24101387 (PMC9611752; doi:10.3390/e24101387)
Supplement: Supplementary file 1 [file entropy-24-01387-s001.zip › Supplementary (SI PDF + Script.zip)/Scripts.zip!/scripts/whole-sum.pdf]

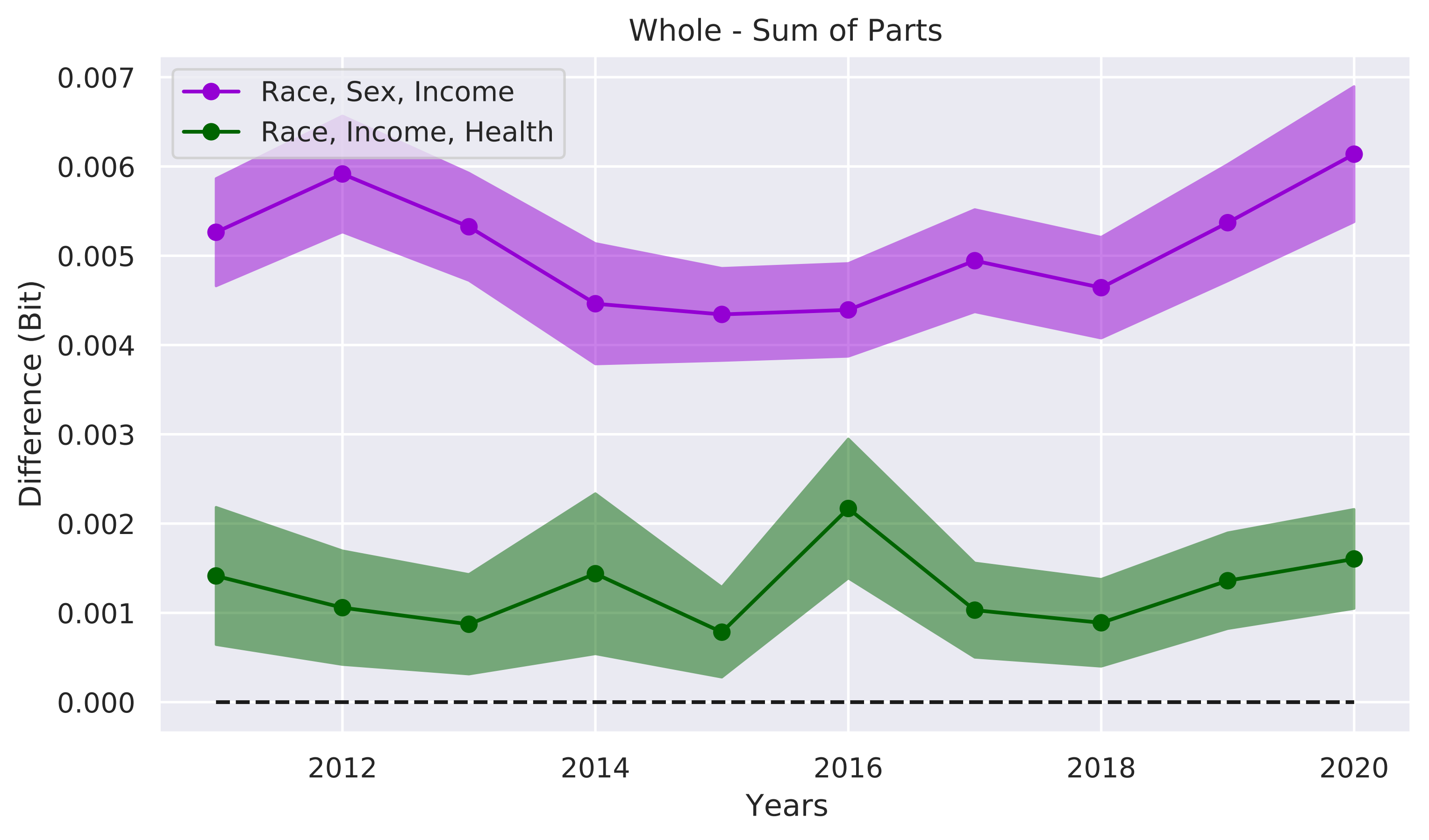
Whole - Sum of Parts
Race, Sex, Income
Race, Income, Health
Difference (Bit)
0.007
0.006
0.005
0.004
0.003
0.002
0.001
0.000
2012
2014
2016
2018
2020
Years
