# Supplementary material for: Untangling Synergistic Effects of Intersecting Social Identities with Partial Information Decomposition
Source: Entropy (Basel). 2022 Sep 28;24(10):1387. doi: 10.3390/e24101387 (PMC9611752; doi:10.3390/e24101387)
Supplement: Supplementary file 1 [file entropy-24-01387-s001.zip › Supplementary (SI PDF + Script.zip)/supplementary_material.zip!/figures/cost-benefit.pdf]

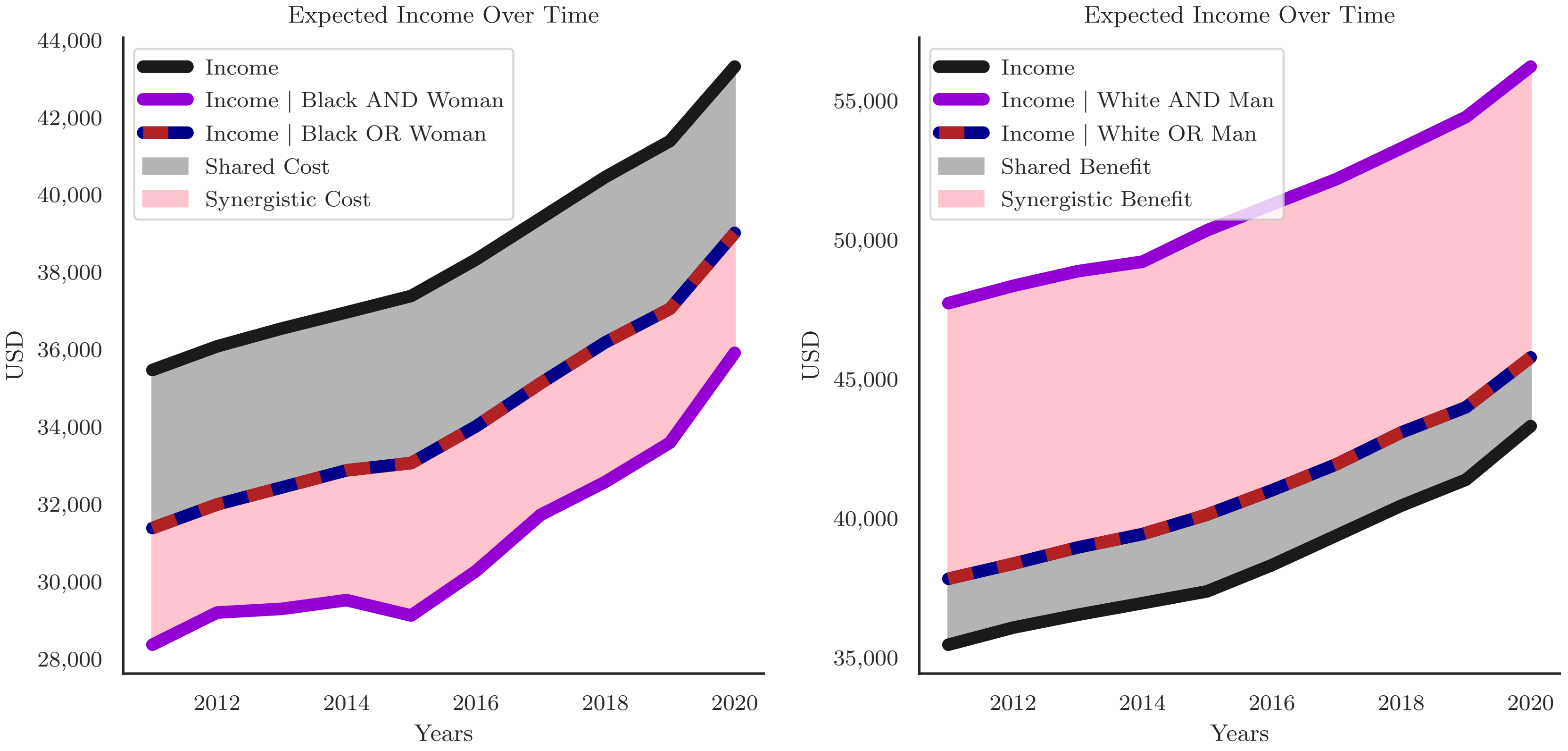
Expected Income Over Time
44,000
42,000
40,000
38,000
36,000
34,000
32,000
30,000
28,000
USD
2012
2014
2016
2018
2020
Years
Income
Income | Black AND Woman
Income | Black OR Woman
Shared Cost
Synergistic Cost
Expected Income Over Time
55,000
50,000
45,000
40,000
35,000
USD
2012
2014
2016
2018
2020
Years
Income
Income | White AND Man
Income | White OR Man
Shared Benefit
Synergistic Benefit
