# Supplementary material for: Untangling Synergistic Effects of Intersecting Social Identities with Partial Information Decomposition
Source: Entropy (Basel). 2022 Sep 28;24(10):1387. doi: 10.3390/e24101387 (PMC9611752; doi:10.3390/e24101387)
Supplement: Supplementary file 1 [file entropy-24-01387-s001.zip › Supplementary (SI PDF + Script.zip)/supplementary_material.zip!/figures/dummies.pdf]

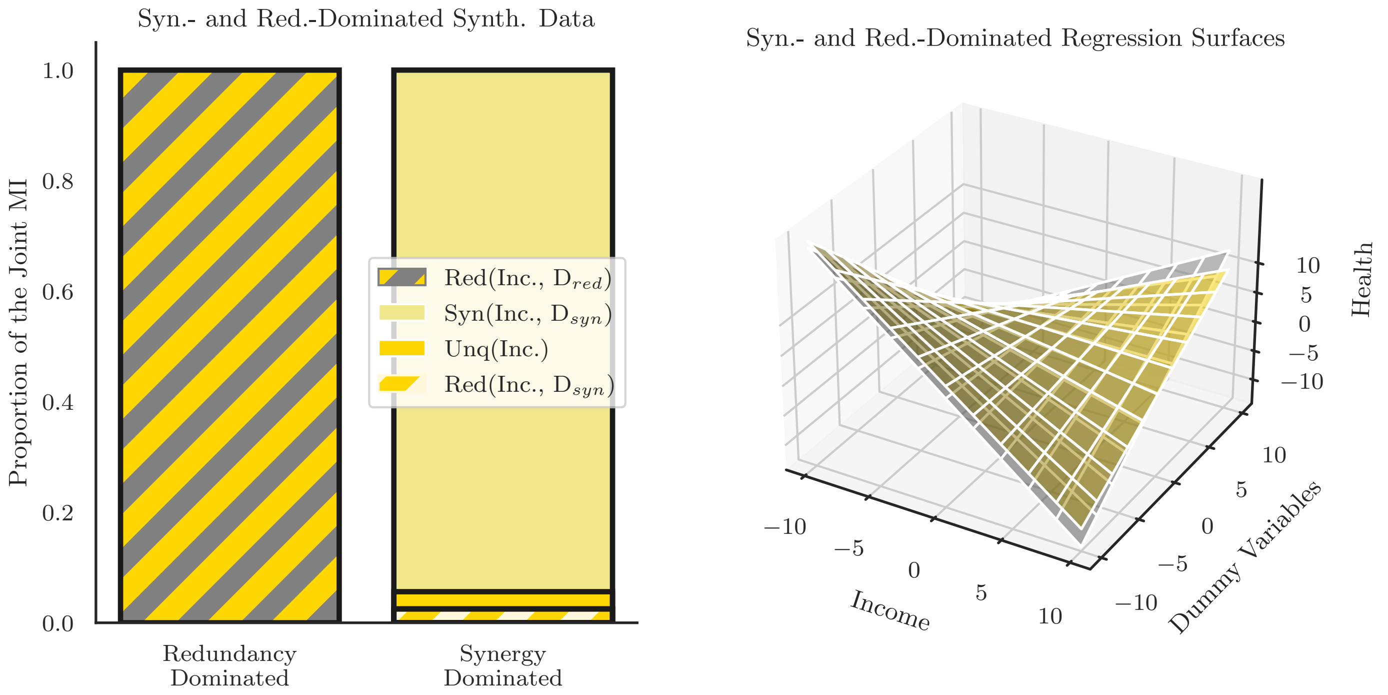
Syn.- and Red.-Dominated Synth. Data
Proportion of the Joint MI
1.0
0.8
0.6
0.4
0.2
0.0
Red(Inc., D_{red})
Syn(Inc., D_{syn})
Unq(Inc.)
Red(Inc., D_{syn})
Redundancy Dominated
Synergy Dominated
Syn.- and Red.-Dominated Regression Surfaces
Health
10
5
0
−5
−10
Income
Dummy Variables
