# Supplementary material for: Untangling Synergistic Effects of Intersecting Social Identities with Partial Information Decomposition
Source: Entropy (Basel). 2022 Sep 28;24(10):1387. doi: 10.3390/e24101387 (PMC9611752; doi:10.3390/e24101387)
Supplement: Supplementary file 1 [file entropy-24-01387-s001.zip › Supplementary (SI PDF + Script.zip)/supplementary_material.zip!/figures/pid_bars.pdf]

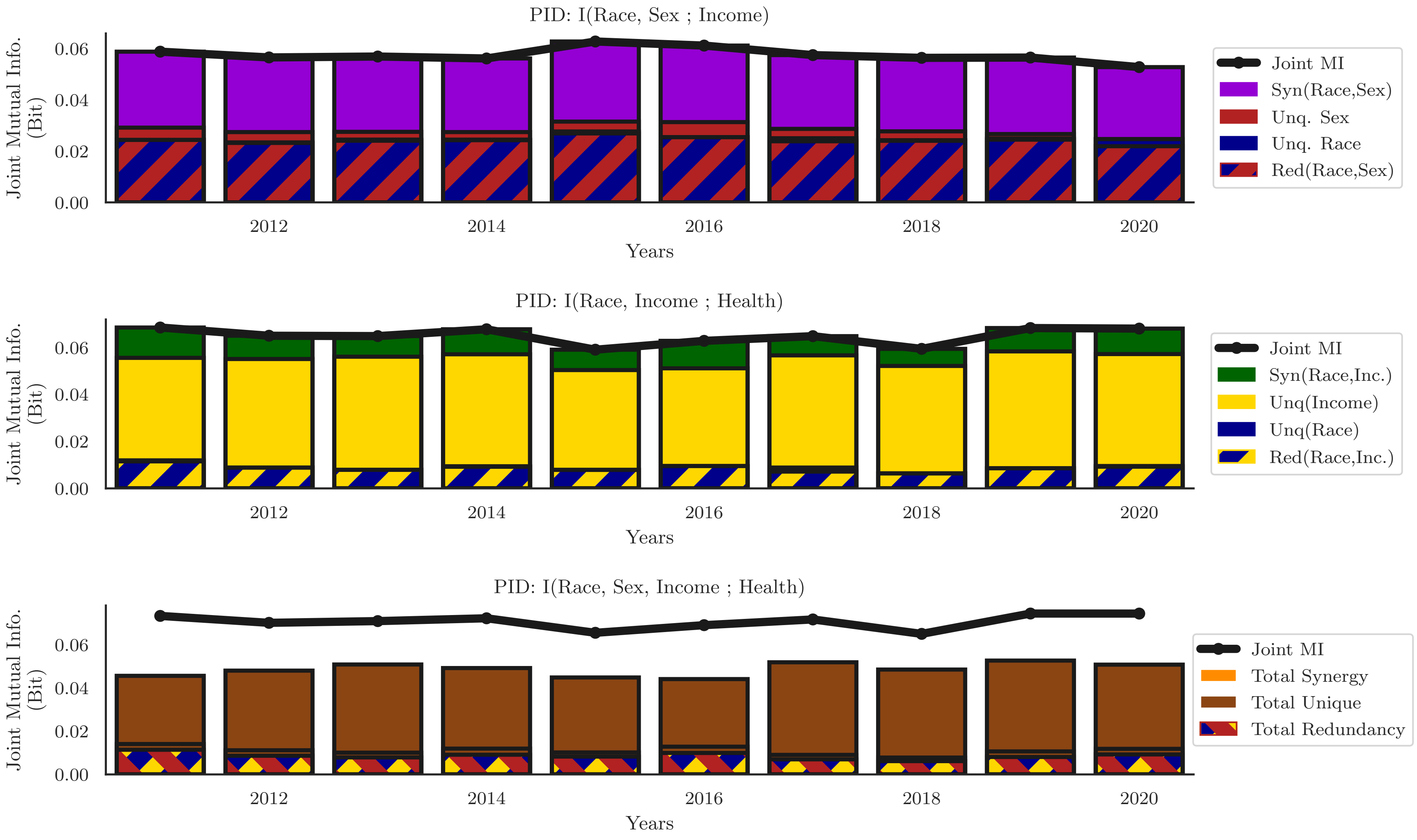
PID: I(Race, Sex ; Income)
Joint Mutual Info. (Bit)
0.00
0.02
0.04
0.06
2012
2014
2016
2018
2020
Years
Joint MI
Syn(Race,Sex)
Unq. Sex
Unq. Race
Red(Race,Sex)
PID: I(Race, Income ; Health)
Joint Mutual Info. (Bit)
0.00
0.02
0.04
0.06
2012
2014
2016
2018
2020
Years
Joint MI
Syn(Race,Inc.)
Unq(Income)
Unq(Race)
Red(Race,Inc.)
PID: I(Race, Sex, Income ; Health)
Joint Mutual Info. (Bit)
0.00
0.02
0.04
0.06
2012
2014
2016
2018
2020
Years
Joint MI
Total Synergy
Total Unique
Total Redundancy
