# Supplementary material for: Untangling Synergistic Effects of Intersecting Social Identities with Partial Information Decomposition
Source: Entropy (Basel). 2022 Sep 28;24(10):1387. doi: 10.3390/e24101387 (PMC9611752; doi:10.3390/e24101387)
Supplement: Supplementary file 1 [file entropy-24-01387-s001.zip › Supplementary (SI PDF + Script.zip)/supplementary_material.zip!/figures/pid_venn.pdf]

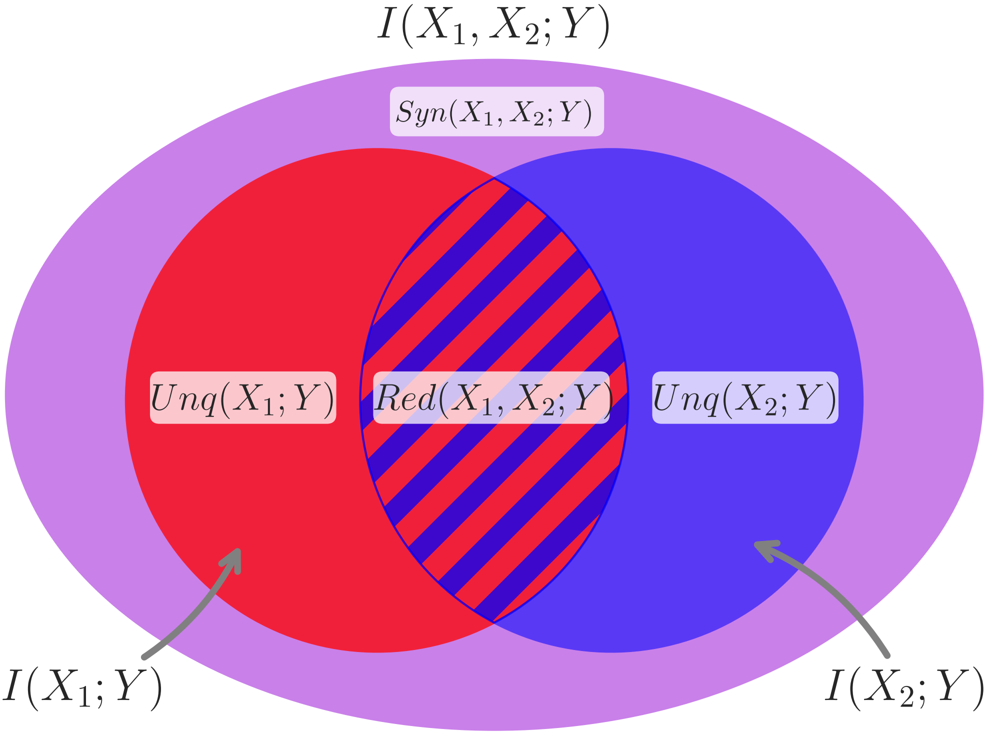
$I(X_1, X_2; Y)$
$Syn(X_1, X_2; Y)$
$Unq(X_1; Y)$
$Red(X_1, X_2; Y)$
$Unq(X_2; Y)$
$I(X_1; Y)$
$I(X_2; Y)$
