# Supplementary material for: Untangling Synergistic Effects of Intersecting Social Identities with Partial Information Decomposition
Source: Entropy (Basel). 2022 Sep 28;24(10):1387. doi: 10.3390/e24101387 (PMC9611752; doi:10.3390/e24101387)
Supplement: Supplementary file 1 [file entropy-24-01387-s001.zip › Supplementary (SI PDF + Script.zip)/supplementary_material.zip!/figures/size_noise_test.pdf]

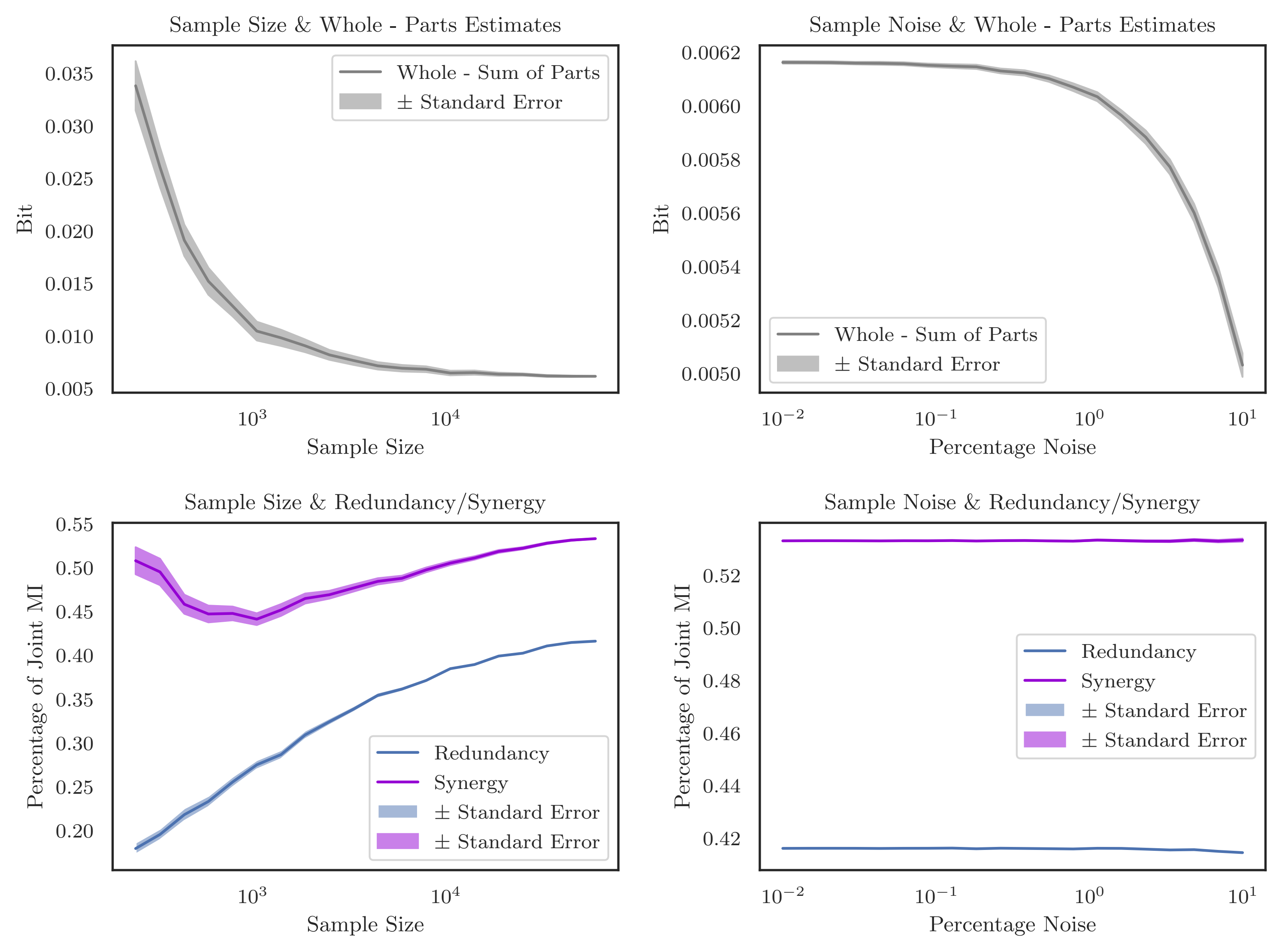
Sample Size & Whole - Parts Estimates
Bit
Whole - Sum of Parts
± Standard Error
Sample Size
Sample Noise & Whole - Parts Estimates
Bit
Whole - Sum of Parts
± Standard Error
Percentage Noise
Sample Size & Redundancy/Synergy
Percentage of Joint MI
Redundancy
Synergy
± Standard Error
± Standard Error
Sample Size
Sample Noise & Redundancy/Synergy
Percentage of Joint MI
Redundancy
Synergy
± Standard Error
± Standard Error
Percentage Noise
