# Supplementary material for: Untangling Synergistic Effects of Intersecting Social Identities with Partial Information Decomposition
Source: Entropy (Basel). 2022 Sep 28;24(10):1387. doi: 10.3390/e24101387 (PMC9611752; doi:10.3390/e24101387)
Supplement: Supplementary file 1 [file entropy-24-01387-s001.zip › Supplementary (SI PDF + Script.zip)/supplementary_material.zip!/supplementary_material.pdf]

Supplementary Information

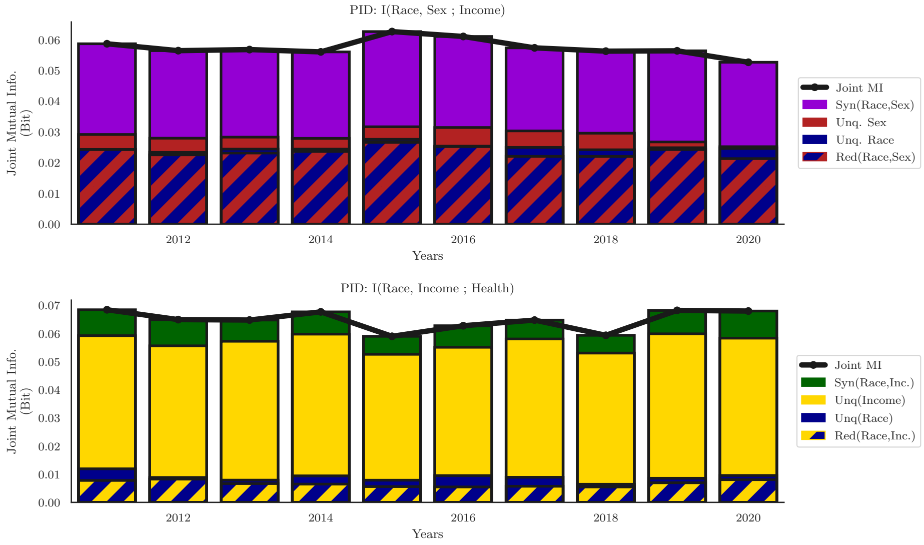

Figure S1: **The PIDs for *I(Race, Sex ; Income)* and *I(Race, Income ; Health)* using the I_{BROJA} function.** We can see that, while the values are not exactly the same, the two different functions for calculating the PIDs (I_{min} from [1] and I_{BROJA} from [2, 3]) find roughly the same distributions of redundant, unique, and synergistic information. This suggests that our analysis is robust to free parameters.

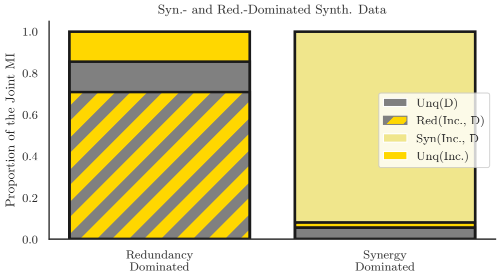

Figure S2: **The PIDs for the synergy and redundancy dominated synthetic datasets using the I_{BROJA} function.** As with the empirical data, the results of the dummy decomposition are functionally the same regardless of whether one is using I_{min} or I_{BROJA}.

References

[1] Paul L. Williams and Randall D. Beer. Nonnegative Decomposition of Multivariate Information. *arXiv:1004.2515 [math-ph, physics:physics, q-bio]*, April 2010. arXiv: 1004.2515.

[2] Nils Bertschinger, Johannes Rauh, Eckehard Olbrich, Jürgen Jost, and Nihat Ay. Quantifying Unique Information. *Entropy*, 16(4):2161–2183, April 2014. Number: 4 Publisher: Multidisciplinary Digital Publishing Institute.

[3] Virgil Griffith and Christof Koch. Quantifying synergistic mutual information. *arXiv:1205.4265 [cs, math, q-bio]*, March 2014. arXiv: 1205.4265.
